# Supplementary material for: Emergent vulnerability to climate-driven disturbances in European forests
Source: Nat Commun. 2021 Feb 23;12:1081. doi: 10.1038/s41467-021-21399-7 (PMC7902618; doi:10.1038/s41467-021-21399-7)
Supplement: Supplementary file 1 — Supplementary Information [file 41467_2021_21399_MOESM1_ESM.pdf]

Emergent vulnerability to climate-driven disturbances in European forests

Giovanni Forzieri, Marco Girardello, Guido Ceccherini, Jonathan Spinoni, Luc Feyen, Henrik Hartmann, Pieter S.A. Beck, Gustau Camps-Valls, Gherado Chirici, Achille Mauri, Alessandro Cescatti

Supplementary Information:

- **Methods 1**
- **Tables 1-3**
- **Figures 1-14**
- **References**

Supplementary Methods 1. Environmental predictors

In order to explore the environmental drivers of forest vulnerability to natural disturbances, data were collected for a set of climate, forest and landscape variables that were used as potential predictors in the vulnerability models. The variables were preliminarily selected based on published studies documenting their potential role in forest disturbances (step2.2 in Supplementary Fig. 1 and discussion in the main text). Selected variables can be static or dynamic. In the former case, one value was considered representative of the entire observational period. In the latter case, a time series with annual time step of the specific environmental variable was used. Dynamic variables can describe the conditions in the year of a given disturbance and/or lagged effects (e.g., anomalies in the years preceding the disturbance).

We browsed for potentially useful datasets and selected the most suitable candidate for each environmental variable based on the following criteria:

- Geographical coverage: global datasets were preferred over national or continental ones in order to avoid inconsistent data across Europe and the United States, which are the spatial domains of the reference disturbance databases.
- Data consistency: datasets with transparent and consistent mapping and reporting methodologies.
- Spatial resolution: highest possible.
- Data update: most recent.

The selected variables and datasets are detailed below, grouped per category: forest, climate and landscape features.

Forest features

- Above ground biomass (biomass). Biomass was derived from multiple Earth Observation data in the framework of the ESA's GlobBiomass project[1]. Data were provided at 100-meter spatial resolution and refer to the year 2010. Data source: https://globbiomass.org/products/global-mapping/. Temporal variations in biomass over the 2000-2017 period were reconstructed by integrating the static biomass map and the forest cover changes derived from the Global Forest Change (GFC) maps recorded at 30-meter spatial resolution from Landsat imagery[2] and available for the 2000-2017 period (see Methods). Data source: https://earthenginepartners.appspot.com/science-2013-global-forest.
- Tree height. Tree height values were retrieved from 1-km spaceborne light detection and ranging (lidar) data acquired in 2005 by the Geoscience Laser Altimeter System (GLAS) aboard ICESat (Ice, Cloud, and land Elevation Satellite)[3]. Data source: https://webmap.ornl.gov/wcsdown/dataset.jsp?ds_id=10023.
- Tree age. Tree age was retrieved from the global forest age dataset (GFAD) describing the age distributions of plant functional types (PFT) on a 0.5-degree grid and represents the 2000-2010 period[4]. The mode of the distribution for each PFT was retrieved and then averaged using PFT-specific cover fractions as weights for RF runs in steps 8-10 (Supplementary Fig. 1). PFT-specific age maps were used for PFT-specific vulnerability models (step11 in Supplementary Fig. 1). Data source: https://doi.pangaea.de/10.1594/PANGAEA.889943.
- Leaf Area Index (LAI). Growing season averages of LAI were retrieved from MODIS Terra+Aqua data provided at 500-meter spatial resolution over the 2002-2017 period (MCD15A3H.006, data source: https://doi.org/10.5067/MODIS/MCD15A3H.006).

The growing season spans from June to September. Missing LAI values for years 2000-2001 were reconstructed from NDVI values by interpolation of a quadratic polynomial fitting function calibrated over the overlapping period 2002-2017. NDVI values were retrieved from MODIS Terra data at 250-meter spatial resolution over the 2000-2017 period (MOD13Q1.006, data source: https://doi.org/10.5067/MODIS/MOD13Q1.006).

- Tree density. Tree densities were retrieved from a database of predictive regression models that link tree density observed over a multitude of plots at global scale with spatially explicit information on climate, topography, vegetation characteristics, and anthropogenic land use[5]. Tree density data were provided as a static map at 1-km spatial resolution and refer to the last two decades. Data source: https://doi.org/10.6084/m9.figshare.3179986.

Climate features

- Annual cumulated precipitation (Pcum), annual average temperature (Tavg) and annual maximum temperature (Tmax). Pcum, Tavg and Tmax were retrieved from the TerraClimate dataset, which combines high-spatial resolution climatological normals from the WorldClim dataset with time-varying coarser data from CRU Ts4.0 and the Japanese 55-year Reanalysis (JRA55)[6]. Pavg, Tavg and Tmax refer to the annual precipitation and temperature conditions during the disturbance year and were provided at ~4-km spatial resolution over the period 1979-2018. Data source: http://www.climatologylab.org/terraclimate.html.
- Short-term average anomaly in cumulated precipitation (avg aPcum) and average temperature (avg aTavg). avg aPcum (avg aTavg) were quantified as the average of the annual anomalies in cumulated precipitation (average temperature) over a six-year time window [t-5,t], where t is the year of the disturbance occurrence. Annual anomalies in cumulated precipitation (average temperature) were computed as the difference between the annual cumulated precipitation (average temperature) and its climatological value over the period 1970-1990. Temperature and precipitation were retrieved from the TerraClimate database[6] at ~4-km spatial resolution over the period 1979-2018. Data source: http://www.climatologylab.org/terraclimate.html.
- Long-term average cumulated precipitation (Long-term Pcum) and long-term average temperature (Long-term Tavg). Long-term Pcum (Long-term Tavg) was quantified as the average of annual cumulated precipitation (average temperature) over the period 1979-2018. Temperature and precipitation are retrieved from the TerraClimate[6] database at ~4-km spatial resolution over the period 1979-2018. Data source: http://www.climatologylab.org/terraclimate.html.
- Annual moisture index (MI). MI was quantified as the minimum of the seasonal MIs in a year, which were derived as (seasonal cumulated precipitation)/(seasonal maximum temperature + 30). The approach is based on a modified version of the De Martonne index[7], where the constant 30 at the denominator is introduced to avoid negative values in cold climates. Temperature and precipitation were retrieved from the TerraClimate[6] database at ~4-km spatial resolution over the period 1979-2018. Data source: http://www.climatologylab.org/terraclimate.html.
- Short-term average standardized precipitation evapotranspiration index (avg SPEI). avg SPEI was quantified as the average of the annual 12-month SPEI computed over a six-year time window [t-5,t], where t is the year of the disturbance occurrence. Monthly SPEI-12 follows the computation approach described in ref. ([8]) and is based

on the difference between precipitation and potential evapotranspiration. In order to characterize prolonged period of water stress conditions before the occurrence of a given disturbance, we first isolated the SPEI-12 ≤ -0.5 values and summed them (in absolute values) over the 12 monthly values. No positive SPEI-12 or just-negative (between -0.5 and 0) values have been included in the annual summed values. Input climate variables were derived from reanalysis data at 0.25° spatial resolution over the period 1979-2018. Data source: https://www.ecmwf.int/en/forecasts/datasets/reanalysis-datasets/era5.

- Fire Weather Index (FWI). The FWI is composed of three moisture codes and three fire behaviour indices. The moisture codes describe the moisture content of three generalized fuel classes, while the behaviour indices represent the spread rate, fuel consumption and intensity of a fire if it were to start[9]. FWI calculations require measurements of temperature at 2m, relative humidity at 2m, and wind speed at 10m, daily snow-depth, and precipitation totaled over the previous 24 hours. FWI was provided from the Global Fire WEather Database (GFWED) at 0.5° spatial resolution over the 1980-2018 period. Fire danger is typically mapped in classes (very low, low, medium, high, very high and extreme) according to FWI values. In our study, we used as predictor the number of days within a year with FWI above the "high danger" level. Data source: https://data.giss.nasa.gov/impacts/gfwed/.
- Annual maximum wind speed (Wind speed) and cumulated snow (Snow). Wind speed and Snow values were retrieved from the NCEP-DOE Reanalysis 2 project which uses a state-of-the-art analysis/forecast system to perform data assimilation[10]. Both annual maximum wind speed and cumulated snow were provided at 0.5° spatial resolution over the 1979-2018 period. Data sources: https://www.esrl.noaa.gov/psd/data/gridded/data.ncep.reanalysis2.html.

Landscape features

- Population density. Human population density depicts the distribution of population, expressed as the number of people per unit surface and has been produced within a framework tested with a large set of sensors including radar and optical public and commercial missions[11]. The original spatial resolution of 250 meter was resampled to 0.25° to better capture features of ignition probability and fire suppression[12,13]. Population density estimates were provided for the years 1975, 1990, 2000 and 2015. Missing years over the 2000-2017 period (RF calibration/validation temporal window) have been retrieved by linear interpolation. Data source: http://ghsl.jrc.ec.europa.eu/ghs_pop.php.
- Coefficient of spatial variation (CV), Evenness Index (Evenness) and Homogeneity Index (Homogeneity). CV, Evenness and Homogeneity are metrics quantifying the spatial heterogeneity of landscape patterns based on the textural features of Enhanced Vegetation Index (EVI) imagery acquired by the Moderate Resolution Imaging Spectroradiometer (MODIS)[14]. Such spatial diversity metrics were provided at 1-km spatial resolution, are static and refer to the 2001-2005 period. Data source: http://www.earthenv.org/texture.
- Elevation and Slope. Elevation and slope describe key geomorphic features and were derived from the Global Multi-resolution Terrain Elevation Data (GMTED2010) provided at a 250 meter spatial resolution. Data source: https://www.usgs.gov/land-resources/eros/coastal-changes-and-impacts/gmted2010.

Category	Full name	Acronym	Fires	Windthrows	Insect outbreaks
Forest	Above ground biomass	Biomass	A,I,Q	A,I,Q	A,I,Q
	Tree height	Tree height	A,	A,I,Q	A,I,Q
	Tree age	Tree age	A,I,Q	A,I,Q	A,I,Q
	Leaf Area Index	LAI	A,I,Q	A,I,Q	A,I,Q
	Tree density	Tree density	A,I,Q	A,I,Q	A,I,Q
Climate	Annual cumulated precipitation	Pcum	A,I,Q	A,I,Q	
	Short-term average anomaly in cumulated precipitation	avg aPcum			A,I,Q
	Annual cumulated snow	Snow		A,I,Q	
	Annual average temperature	Tavg	A,I		
	Short-term average anomaly in average temperature	avg aTavg			A,I,Q
	Annual maximum temperature	Tmax	A,I,Q		
	Annual aridity index	MI	A,I,Q		
	Short-term average standardized precipitation evapotranspiration index	avg SPEI			A,I,Q
	Fire Weather Index	FWI	A,I,Q		
	Annual maximum wind speed	Wind speed		A,I,Q	
	Long-term average cumulated precipitation	Long-term Pavg	A	A,I,Q	A
	Long-term average temperature	Long-term Tavg	A	A	A,I,Q
Landscape	Population density	Population	A,I,		
	Coefficient of spatial variation	CV	A		A,I,Q
	Evenness	Evenness	A		A
	Homogeneity	Homogeneity	A,I,Q	A,I,Q	A
	Slope	Slope	A,I,Q	A,I,Q	
	Elevation	Elevation	A		A,I,Q

Table 1. Environmental predictors used in vulnerability models. Variables labelled with "A" are those used as initial input of the first "approximate" vulnerability model (Supplementary Fig. 1, step2.2). Variables labelled with "I" are those that passed the intermediate screening process based on their relative importance (Supplementary Fig. 1, step9). Variables labelled with "Q" are those selected as optimal features to predict the observed vulnerability and used as predictors in the final models (Supplementary Fig. 1, step10).

Disturbance type	Region	Potential relative biomass loss [%]						Potential absolute biomass loss [t ha^{-1}]					
		Avg		ci_min		ci_max		Avg		ci_min		ci_max	
		Whole	Sampled	Whole	Sampled	Whole	Sampled	Whole	Sampled	Whole	Sampled	Whole	Sampled
**Fires**	**Eastern Europe (EEA)**	23.9	23.9	22.6	23.0	24.5	25.0	15.2	15.3	14.6	14.7	15.6	16.0
	**Northern Europe (NEU)**	25.1	26.3	24.0	25.2	25.7	27.5	12.7	15.9	11.9	15.0	12.9	16.8
	**Southern Europe (SEU)**	26.1	26.3	24.4	24.9	26.6	27.2	12.5	12.5	11.6	11.9	12.8	13.3
	**Western Europe (WEU)**	23.4	23.6	22.2	22.5	24.5	24.9	18.0	18.0	17.0	16.9	18.9	19.0
	**European Russia (EUR)**	26.8	27.0	26.1	25.6	27.3	27.5	19.2	20.8	18.5	20.1	19.5	21.3
	**Europe (EU+)**	25.6	25.9	25.0	25.4	25.8	26.3	16.3	17.5	15.9	17.0	16.4	17.8
**Windthrows**	**Eastern Europe (EEA)**	27.0	27.0	25.6	25.9	27.8	28.4	17.4	17.7	16.4	16.8	18.0	18.5
	**Northern Europe (NEU)**	31.2	28.2	29.4	26.7	31.5	29.9	14.5	18.9	13.8	17.5	14.7	20.0
	**Southern Europe (SEU)**	33.5	34.7	31.0	32.5	34.2	37.6	17.1	22.1	15.9	20.6	17.6	23.7
	**Western Europe (WEU)**	31.3	30.7	29.4	28.9	32.8	32.4	24.6	24.7	22.8	22.9	25.7	26.3
	**European Russia (EUR)**	29.9	29.4	29.0	27.7	30.3	31.1	21.2	23.2	20.4	21.3	21.6	24.3
	**Europe (EU+)**	30.2	29.2	29.4	28.4	30.3	29.9	18.9	20.7	18.3	20.0	19.0	21.3
**Insect outbreaks**	**Eastern Europe (EEA)**	18.6	18.4	17.6	17.6	19.0	19.2	11.3	11.4	10.7	10.9	11.7	12.0
	**Northern Europe (NEU)**	20.5	20.5	19.4	19.8	20.9	21.4	9.8	11.3	9.3	10.9	10.1	11.8
	**Southern Europe (SEU)**	19.4	19.0	18.2	17.8	19.8	21.0	9.3	10.7	8.7	10.1	9.7	11.4
	**Western Europe (WEU)**	19.8	19.4	18.7	18.2	20.5	20.6	14.6	14.7	13.5	13.7	15.1	15.6
	**European Russia (EUR)**	20.2	19.8	19.5	19.2	20.5	20.2	13.7	13.8	13.3	13.3	13.9	14.0
	**Europe (EU+)**	19.9	19.7	19.4	19.4	20.0	20.1	12.1	12.7	11.8	12.5	12.2	12.9
**Overall Vulnerability Index**	**Eastern Europe (EEA)**	54.7	54.8	52.2	51.9	56.1	58.5	34.6	35.8	32.8	33.8	35.6	38.0
	**Northern Europe (NEU)**	59.0	55.9	56.4	51.6	61.1	60.8	28.5	38.3	27.0	35.1	29.2	41.4
	**Southern Europe (SEU)**	60.5	59.2	55.9	54.8	61.5	64.9	30.0	38.5	27.9	34.9	30.6	42.2
	**Western Europe (WEU)**	57.7	57.1	54.3	53.9	59.4	60.7	44.3	45.2	41.9	42.1	46.7	48.8
	**European Russia (EUR)**	59.0	56.6	57.1	52.7	59.9	60.3	41.5	45.2	39.9	40.7	42.0	48.4
	**Europe (EU+)**	58.4	56.2	57.0	54.1	58.4	57.6	36.5	39.9	35.5	38.5	36.6	41.0

Table 2. Statistics of current vulnerability of European forest to natural disturbances. Values aggregated per macro-regions and for the full spatial domain (EU+). Metrics include spatial average (Avg) and its 95% confidence interval (ci_min and ci_max) expressed both in relative and absolute biomass loss. Macro-regions are shown in Supplementary Fig. 14. The “Whole” field refers to estimates derived from the whole domain of European forests, whereas the field “Sampled” refers to the estimates derived from areas within the climatological ranges of the observational sets of natural disturbances (Supplementary Fig. 8).

Disturbance type	Region	Relative biomass loss [% $year^{-1}$]						Absolute biomass loss [t ha^{-1} $year^{-1}$]					
		Avg		ci_min		ci_max		Avg		ci_min		ci_max	
		**Whole**	**Sampled**	**Whole**	**Sampled**	**Whole**	**Sampled**	**Whole**	**Sampled**	**Whole**	**Sampled**	**Whole**	**Sampled**
**Fires**	**Eastern Europe (EEA)**	-6.6E-03	-7.1E-03	-7.1E-03	-7.8E-03	-5.9E-03	-6.5E-03	-4.8E-03	-5.2E-03	-5.4E-03	-5.7E-03	-4.5E-03	-4.7E-03
	**Northern Europe (NEU)**	-8.7E-03	-7.8E-03	-9.4E-03	-8.9E-03	-8.2E-03	-7.2E-03	-3.4E-03	-3.8E-03	-3.7E-03	-4.2E-03	-3.2E-03	-3.4E-03
	**Southern Europe (SEU)**	-4.7E-04	4.0E-05	-1.8E-03	-1.5E-03	1.2E-03	1.5E-03	-7.9E-04	-7.8E-04	-1.4E-03	-1.5E-03	-3.4E-05	1.1E-04
	**Western Europe (WEU)**	1.1E-02	1.1E-02	9.6E-03	1.0E-02	1.1E-02	1.3E-02	7.4E-03	7.9E-03	6.8E-03	6.7E-03	8.1E-03	8.7E-03
	**European Russia (EUR)**	-6.1E-03	-5.9E-03	-6.5E-03	-6.4E-03	-5.8E-03	-5.5E-03	-4.1E-03	-4.1E-03	-4.5E-03	-4.6E-03	-3.8E-03	-3.8E-03
	**Europe (EU+)**	-4.9E-03	-3.9E-03	-5.2E-03	-4.3E-03	-4.6E-03	-3.5E-03	-2.8E-03	-2.5E-03	-3.0E-03	-2.7E-03	-2.6E-03	-2.3E-03
**Windthrows**	**Eastern Europe (EEA)**	-2.4E-03	-2.8E-03	-2.8E-03	-3.3E-03	-1.8E-03	-2.3E-03	-1.5E-03	-1.9E-03	-1.8E-03	-2.2E-03	-1.2E-03	-1.5E-03
	**Northern Europe (NEU)**	-2.3E-04	1.8E-03	-9.9E-04	-1.8E-04	8.2E-04	4.1E-03	3.0E-05	1.4E-03	-3.5E-04	-2.5E-06	4.2E-04	2.6E-03
	**Southern Europe (SEU)**	4.0E-04	2.9E-03	-7.2E-04	2.6E-04	1.4E-03	5.5E-03	6.9E-04	2.5E-03	-3.8E-04	6.4E-04	1.3E-03	4.2E-03
	**Western Europe (WEU)**	-9.9E-03	-1.1E-02	-1.1E-02	-1.2E-02	-8.6E-03	-9.2E-03	-7.4E-03	-8.4E-03	-8.6E-03	-1.0E-02	-6.3E-03	-7.1E-03
	**European Russia (EUR)**	-5.3E-04	1.5E-03	-7.4E-04	9.7E-04	-3.2E-04	1.9E-03	-9.3E-04	9.2E-04	-1.1E-03	5.6E-04	-8.2E-04	1.4E-03
	**Europe (EU+)**	-1.4E-03	-2.2E-03	-1.6E-03	-2.7E-03	-1.1E-03	-1.6E-03	-1.1E-03	-1.6E-03	-1.3E-03	-2.0E-03	-9.3E-04	-1.0E-03
**Insect outbreaks**	**Eastern Europe (EEA)**	7.0E-02	6.4E-02	6.7E-02	6.1E-02	7.3E-02	6.8E-02	3.8E-02	3.6E-02	3.6E-02	3.4E-02	3.9E-02	3.8E-02
	**Northern Europe (NEU)**	1.1E-01	1.1E-01	1.1E-01	1.1E-01	1.1E-01	1.2E-01	4.6E-02	5.4E-02	4.4E-02	5.2E-02	4.8E-02	5.7E-02
	**Southern Europe (SEU)**	7.9E-02	7.3E-02	7.6E-02	6.8E-02	8.3E-02	7.9E-02	3.4E-02	3.7E-02	3.2E-02	3.4E-02	3.6E-02	4.1E-02
	**Western Europe (WEU)**	8.9E-02	8.7E-02	8.4E-02	7.9E-02	9.7E-02	9.3E-02	5.8E-02	5.8E-02	5.3E-02	5.5E-02	6.1E-02	6.3E-02
	**European Russia (EUR)**	8.5E-02	7.9E-02	8.3E-02	7.6E-02	8.7E-02	8.1E-02	4.7E-02	4.4E-02	4.5E-02	4.3E-02	4.8E-02	4.6E-02
	**Europe (EU+)**	8.8E-02	8.4E-02	8.7E-02	8.3E-02	9.0E-02	8.6E-02	4.5E-02	4.6E-02	4.4E-02	4.5E-02	4.6E-02	4.7E-02
**Overall Vulnerability Index**	**Eastern Europe (EEA)**	3.4E-02	2.8E-02	3.2E-02	2.6E-02	3.5E-02	3.0E-02	1.7E-02	1.5E-02	1.7E-02	1.4E-02	1.8E-02	1.6E-02
	**Northern Europe (NEU)**	5.1E-02	2.3E-02	4.9E-02	2.0E-02	5.3E-02	2.6E-02	2.2E-02	1.4E-02	2.1E-02	1.2E-02	2.3E-02	1.6E-02
	**Southern Europe (SEU)**	3.8E-02	3.7E-02	3.6E-02	3.3E-02	4.1E-02	4.3E-02	1.7E-02	2.2E-02	1.6E-02	1.8E-02	1.8E-02	2.4E-02
	**Western Europe (WEU)**	4.7E-02	4.4E-02	4.5E-02	4.0E-02	5.0E-02	4.8E-02	3.0E-02	2.8E-02	2.8E-02	2.6E-02	3.2E-02	3.1E-02
	**European Russia (EUR)**	4.0E-02	3.0E-02	3.9E-02	2.8E-02	4.1E-02	3.3E-02	2.1E-02	2.2E-02	2.0E-02	2.0E-02	2.2E-02	2.4E-02
	**Europe (EU+)**	4.2E-02	3.2E-02	4.1E-02	3.0E-02	4.3E-02	3.3E-02	2.1E-02	1.9E-02	2.0E-02	1.9E-02	2.1E-02	2.0E-02

Table 3. Statistics of trends in vulnerability of European forest to natural disturbances. Values aggregated per macro-regions and for the full spatial domain (EU+). Metrics include spatial average (Avg) and its 95% confidence interval (ci_min and ci_max) expressed both in relative and absolute biomass loss. Macro-regions are shown in Supplementary Fig. 14. The "Whole" field refers to estimates derived from the whole domain of European forests, whereas the field "Sampled" refers to the estimates derived from areas within the climatological ranges of the observational sets of natural disturbances (Supplementary Fig. 8).

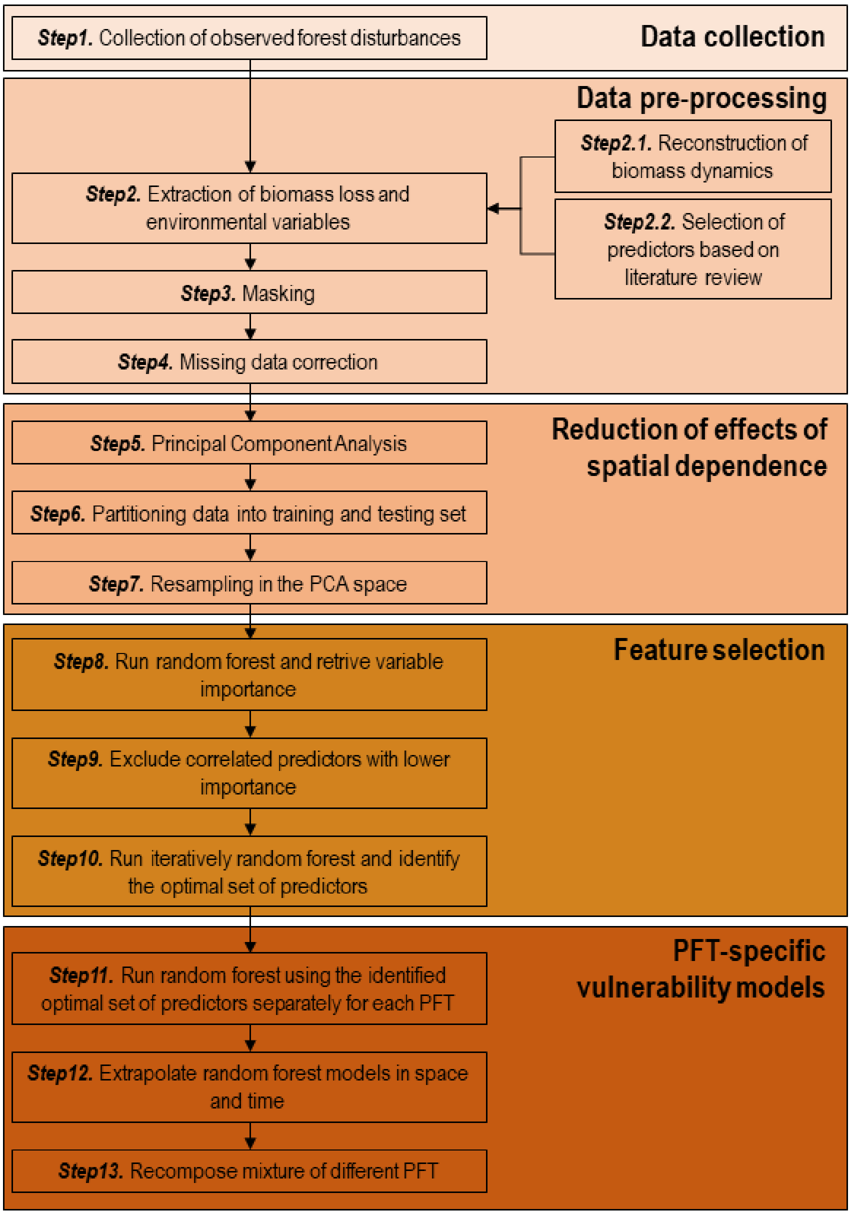

Figure 1. Workflow adopted for the vulnerability models.

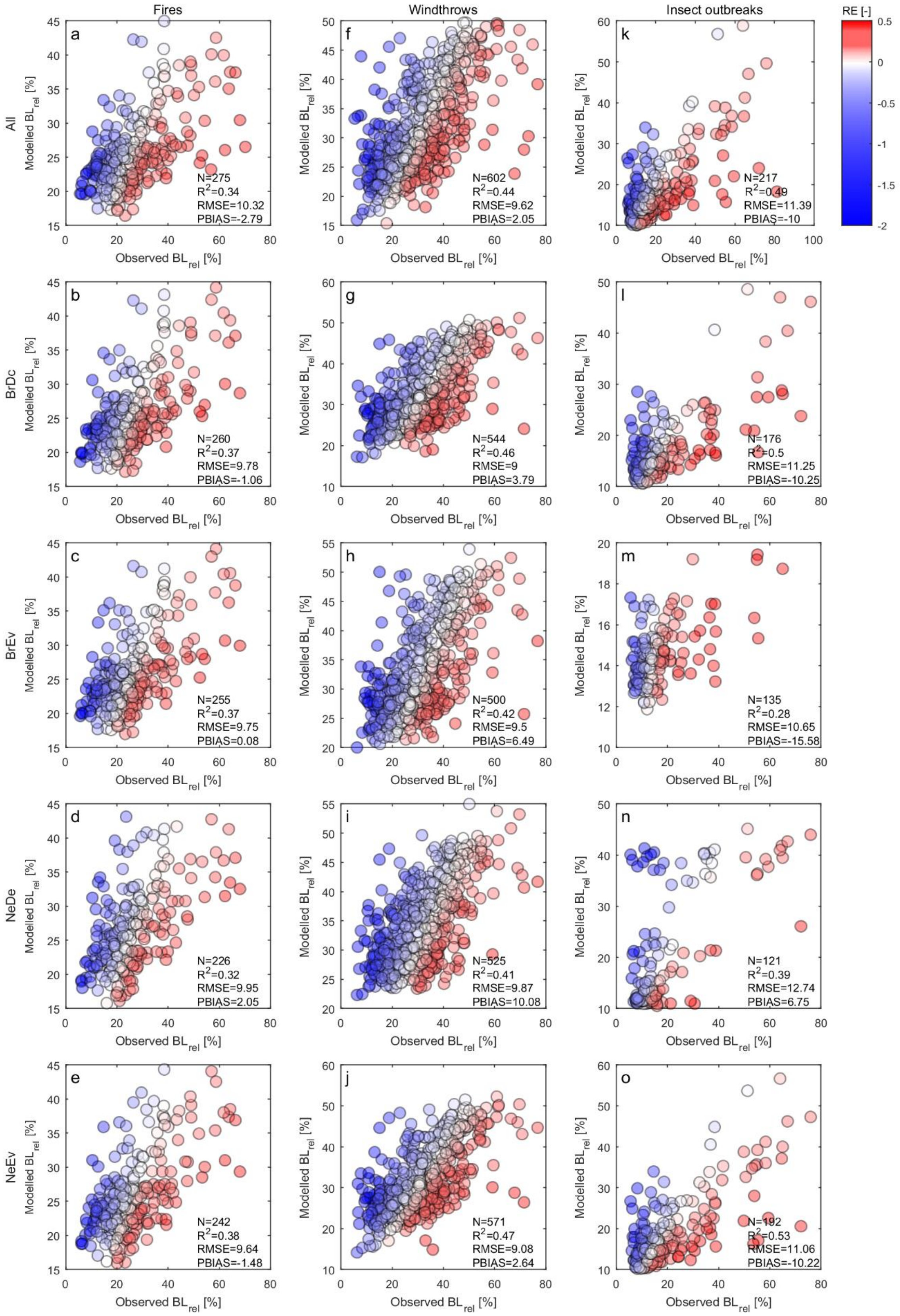

Figure 2. Validation of vulnerability models. Observed versus modelled relative biomass losses (BL_{rel}) for each natural disturbance and plant functional types (PFTs). 'All' refer to the model accounting for the mixture of different PFTs. Number of binned records (N), coefficient of determination (R^2), root mean squared error (RMSE) and percent bias (PBIAS) are shown in labels, while relative error (RE) in colour.

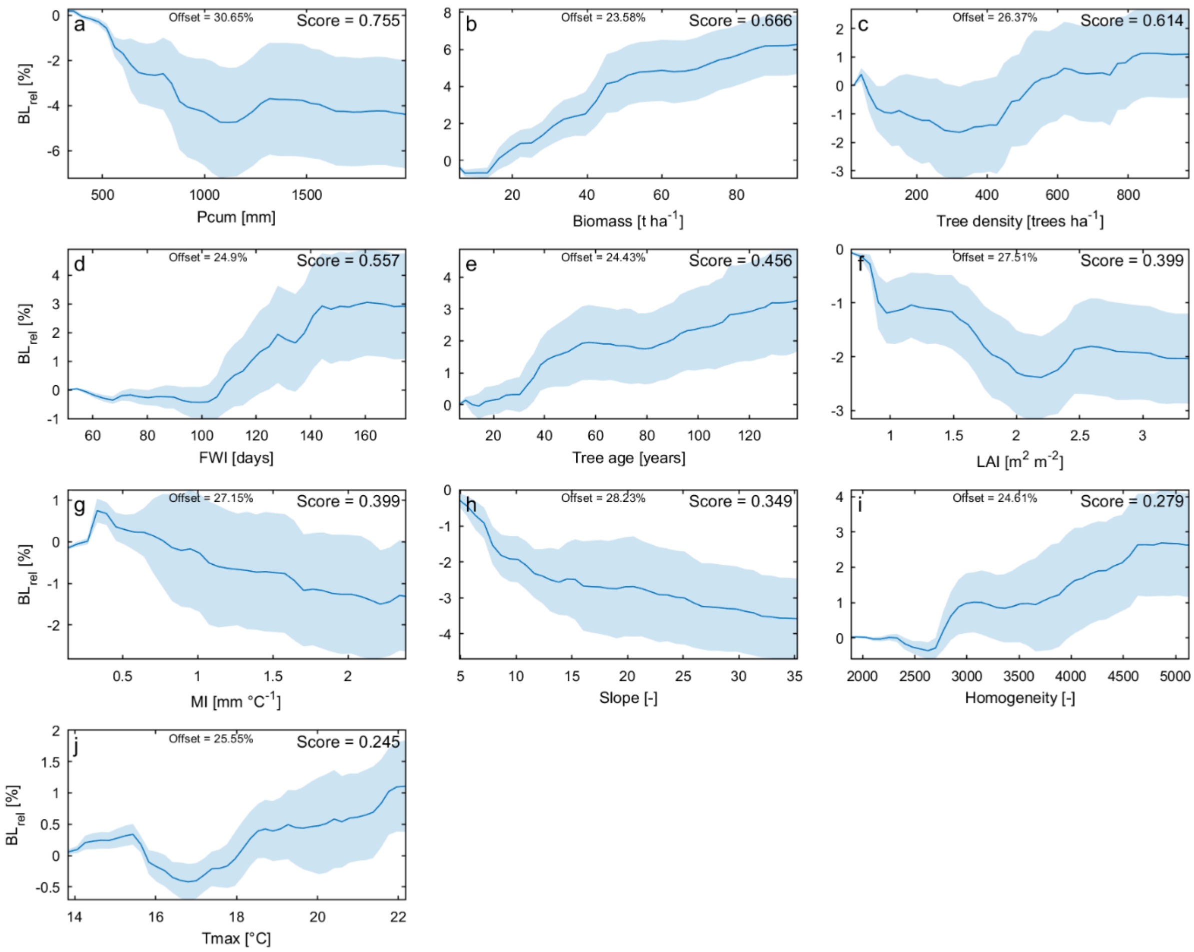

Figure 3. Response functions for forest vulnerability to fires. Zero-centered partial dependence plots (average ± median absolute deviation) showing the dependence of relative biomass loss (BL_{rel} response variable, on the y-axis) on each selected environmental feature (predictor, on the x-axis). Values of environmental predictors span the range between the 0.01 and 0.99 percentiles of the actual distributions. Score values report the variable importance of each predictor and panels are organized in descending order based on their score. Offset values are shown in label for each predictor. Predictor acronyms are listed in Table 1.

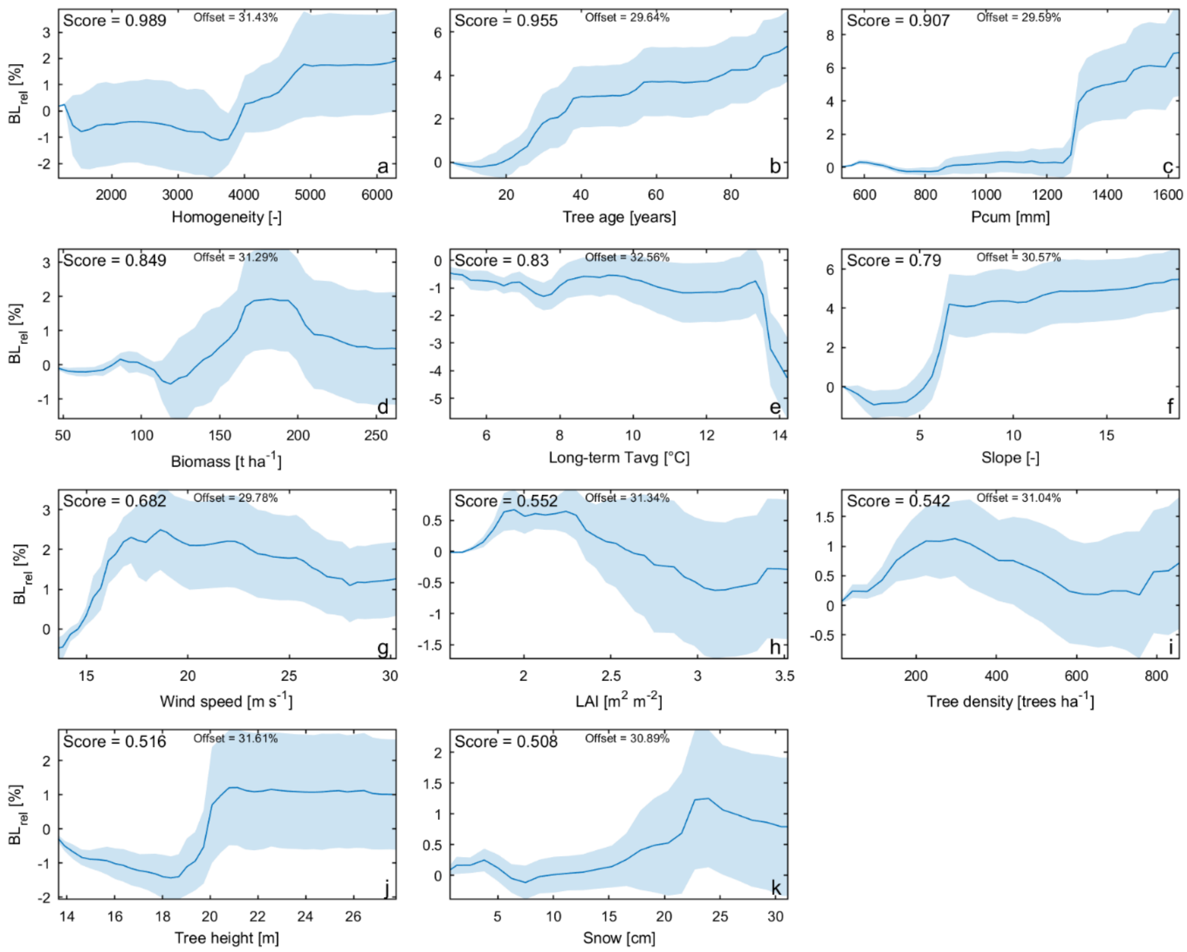

Figure 4. Response functions for forest vulnerability to windthrows. As Supplementary Fig. 3 but for windthrows.

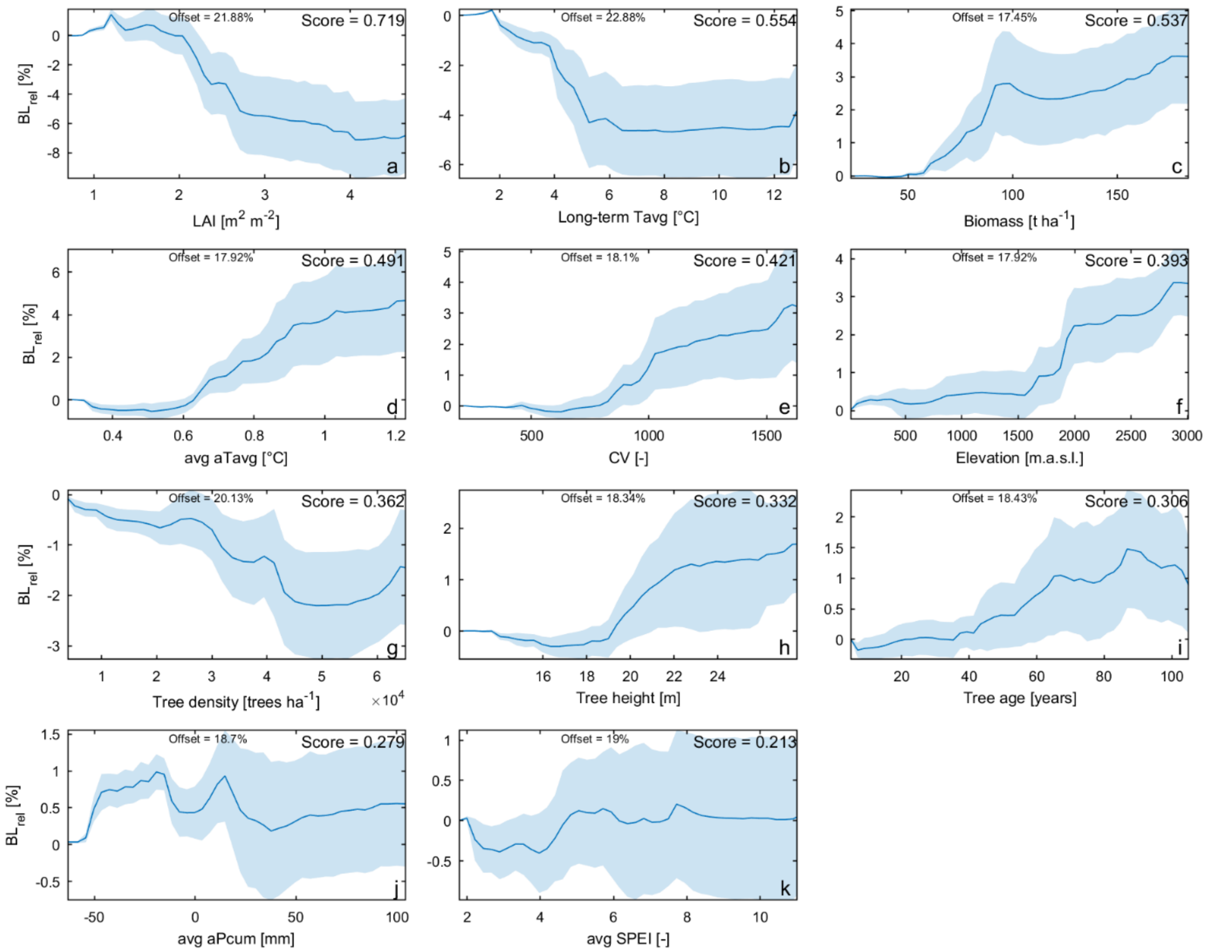

Figure 5. Response functions for forest vulnerability to insect outbreaks. As Supplementary Fig. 3 but for insect outbreaks.

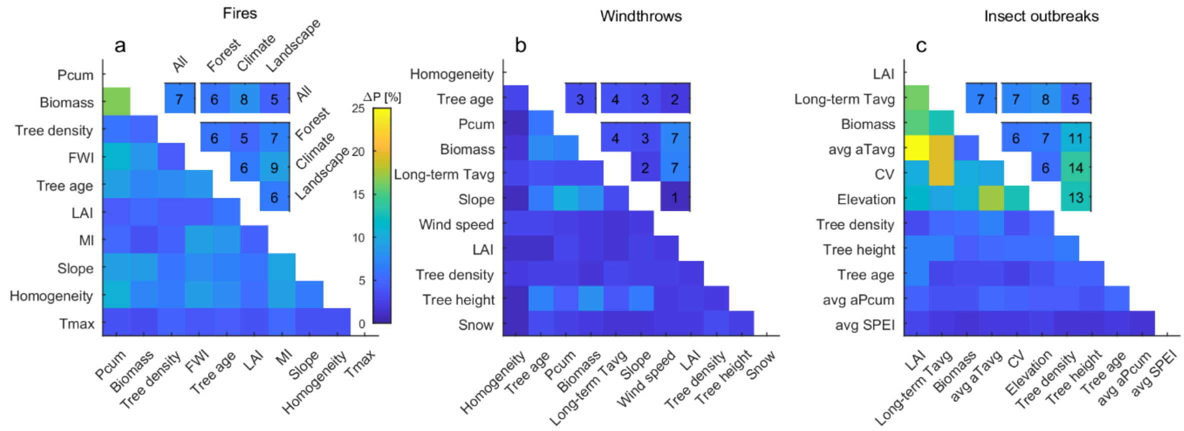

Figure 6. Amplification effects of feature interactions. (**a**) Amplification effect (ΔP) in the peak values of the response functions of forest vulnerability to fires due to second-order interactions amongst environmental predictors (compare to Fig. 3). Averaged values for different combinations of predictor categories (forest, climate, landscape) and for the whole set of features (‘All’) are shown in the inset box (reported in colour and numbers). (**b**) and (**c**) as (**a**) but for vulnerability to windthrows and insect outbreaks, respectively. Predictor acronyms are listed in Table 1.

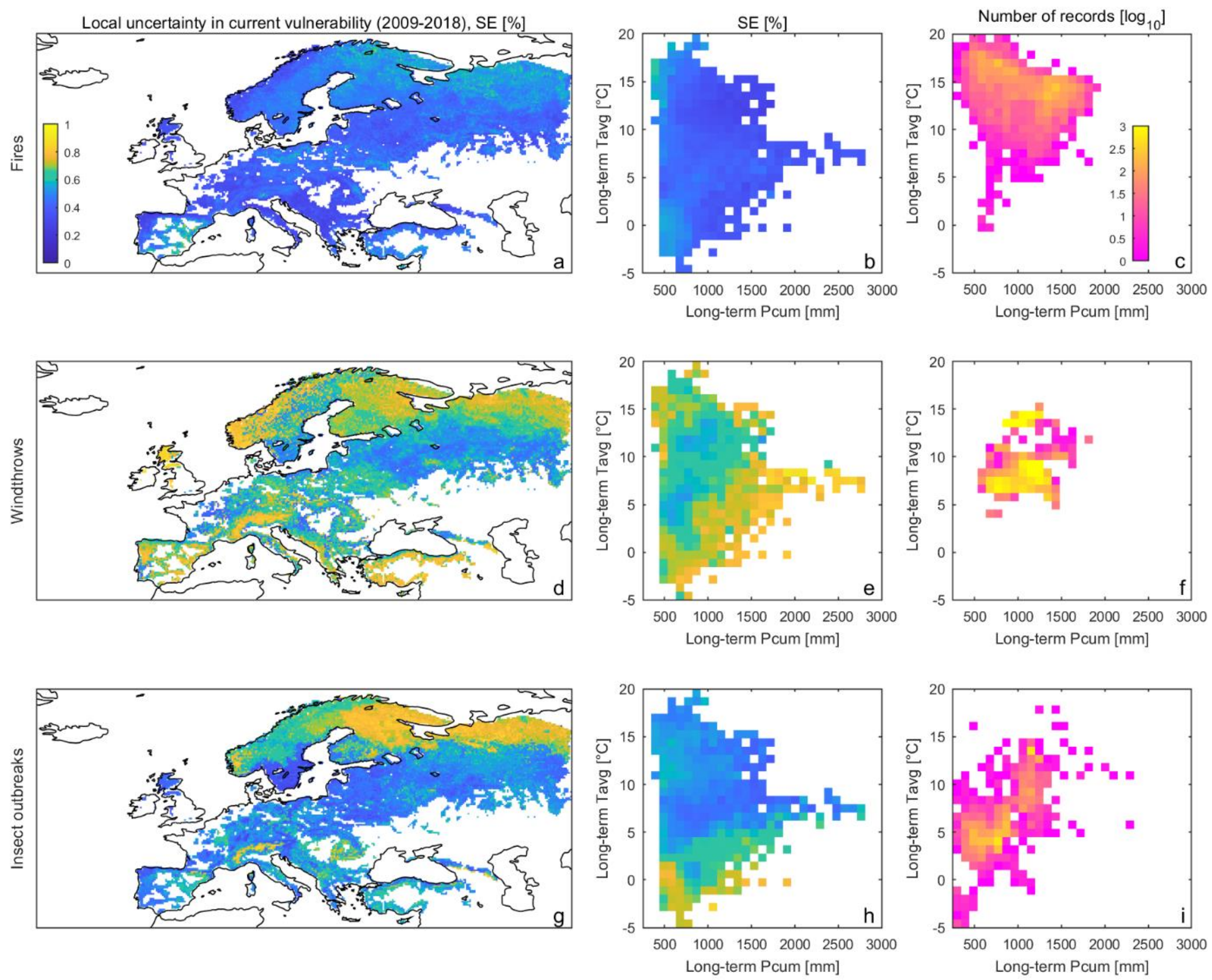

Figure 7. Local uncertainty in current vulnerability. (**a**) Standard error (SE) of current vulnerability of European forests to fires (averaged over the 2009-2018 period). (**b**) SE binned as a function of the long-term cumulated precipitation (on the x-axis) and average temperature (on the y-axis). (**c**) Log-transformed frequency distribution of original forest disturbance records across long-term cumulated precipitation (on the x-axis) and average temperature (on the y-axis). (**d**,**e**,**f**) and (**g**,**h**,**i**) as (**a**,**b**,**c**) but for windthrows and insect outbreaks, respectively.

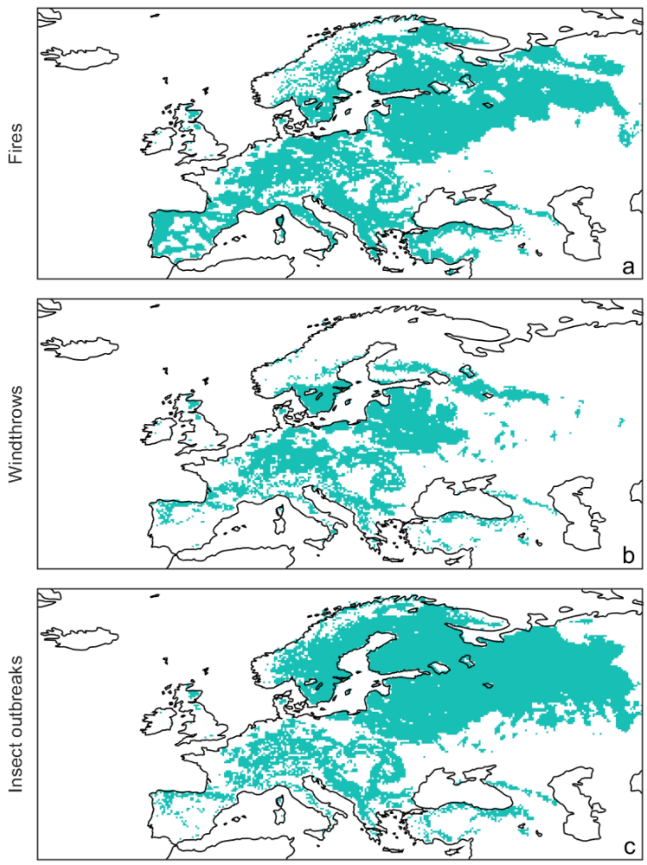

Figure 8. Climate domains sampled by the forest disturbance databases. (**a**) Spatial domain with climatological temperature and precipitation within the observational ranges of fire disturbance records. (**b**) and (**c**) as (**a**) but for windthrows and insect outbreaks, respectively.

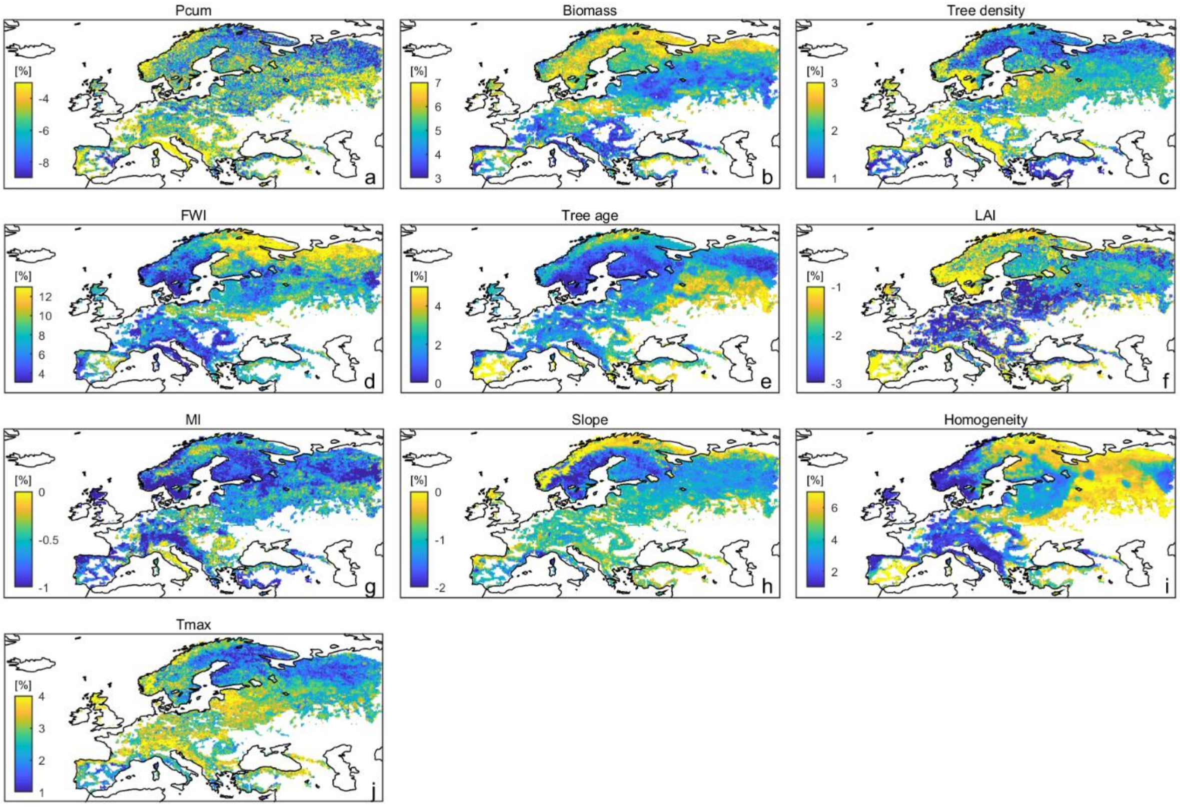

Figure 9. Local sensitivity of forest vulnerability to fires. Sensitivity of vulnerability to fires computed as first-order derivative of the individual conditional expectation retrieved at grid cell level, separately for each environmental predictor. Forests with cover fraction lower than 0.1 are masked in white. Predictor acronyms are listed in Table 1.

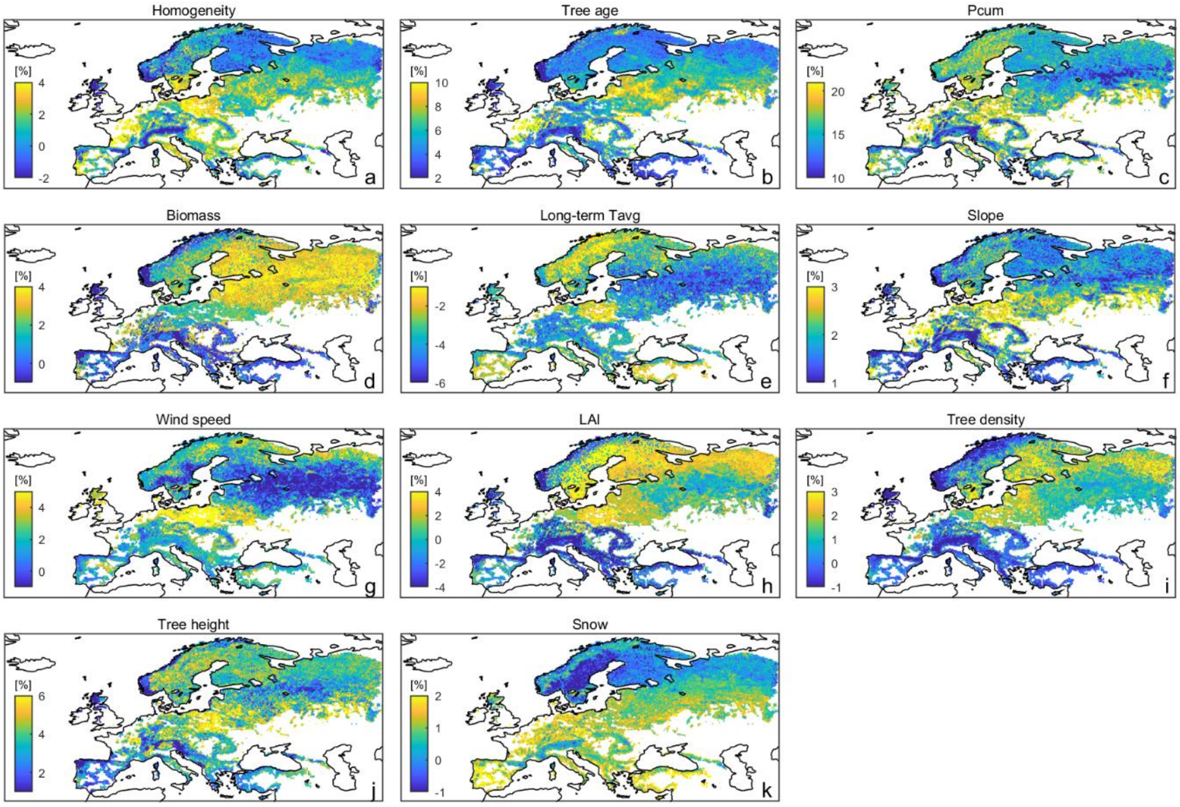

Figure 10. Local sensitivity of forest vulnerability to windthrows. As Supplementary Fig. 9 but for windthrows.

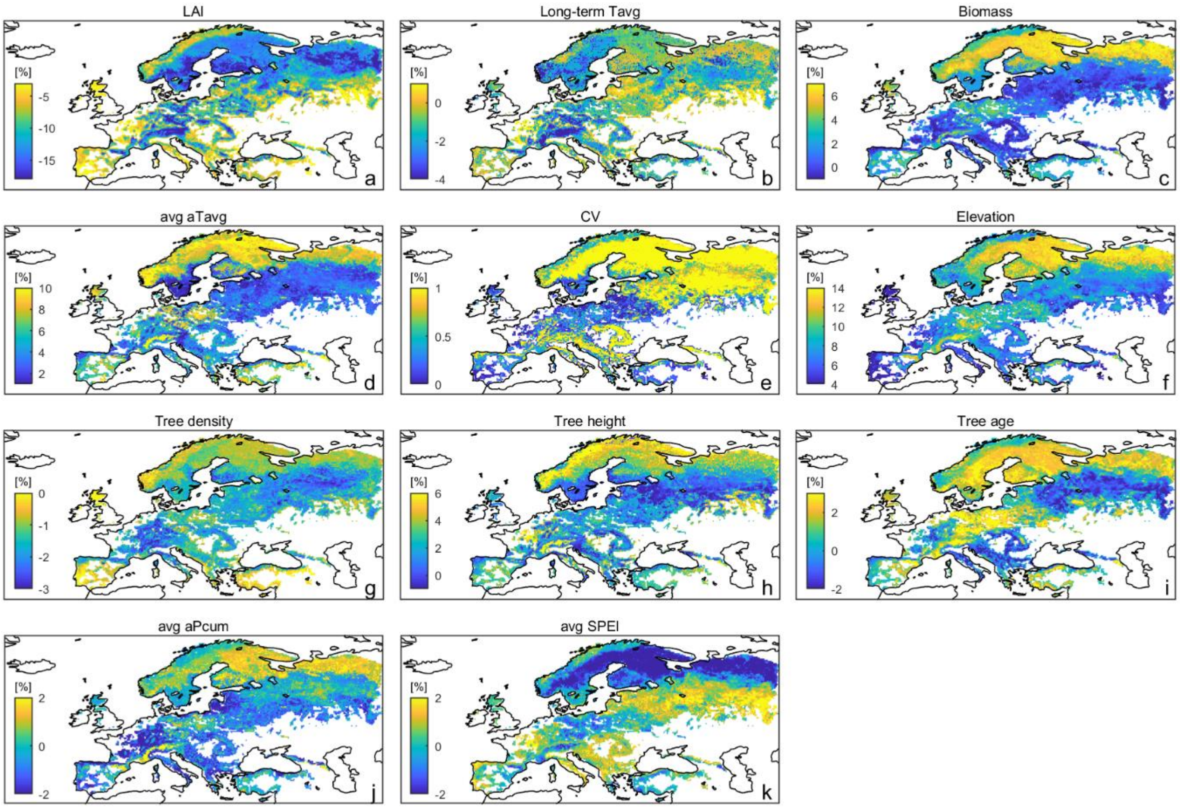

Figure 11. Local sensitivity of forest vulnerability to insect outbreaks. As Supplementary Fig. 9 but for insect outbreaks.

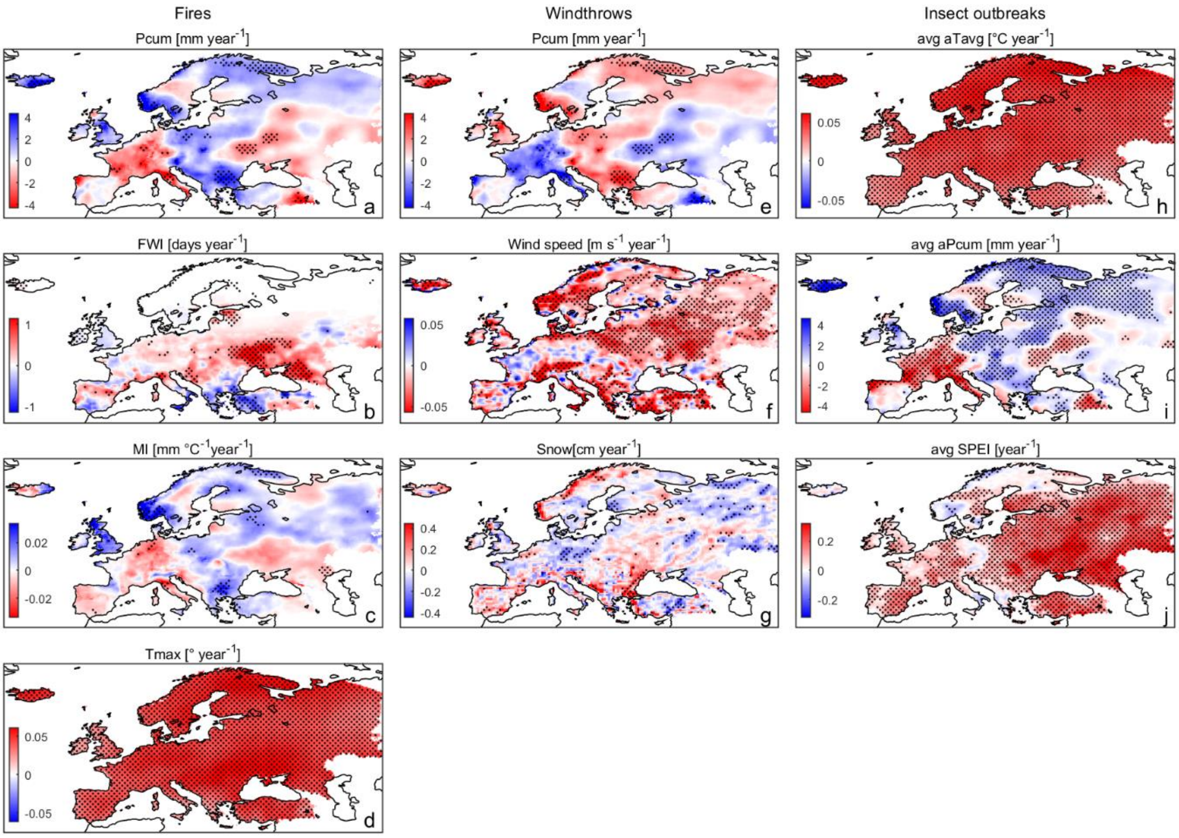

Figure 12. Trends in climate drivers of forest vulnerability to natural disturbances. Temporal trends in climate drivers (1979-2018). Black dots show pixels where trends are significant (two-sided Mann-Kendall test; p-value<0.05). Maps are grouped per natural disturbance. Predictor acronyms are listed in Table 1.

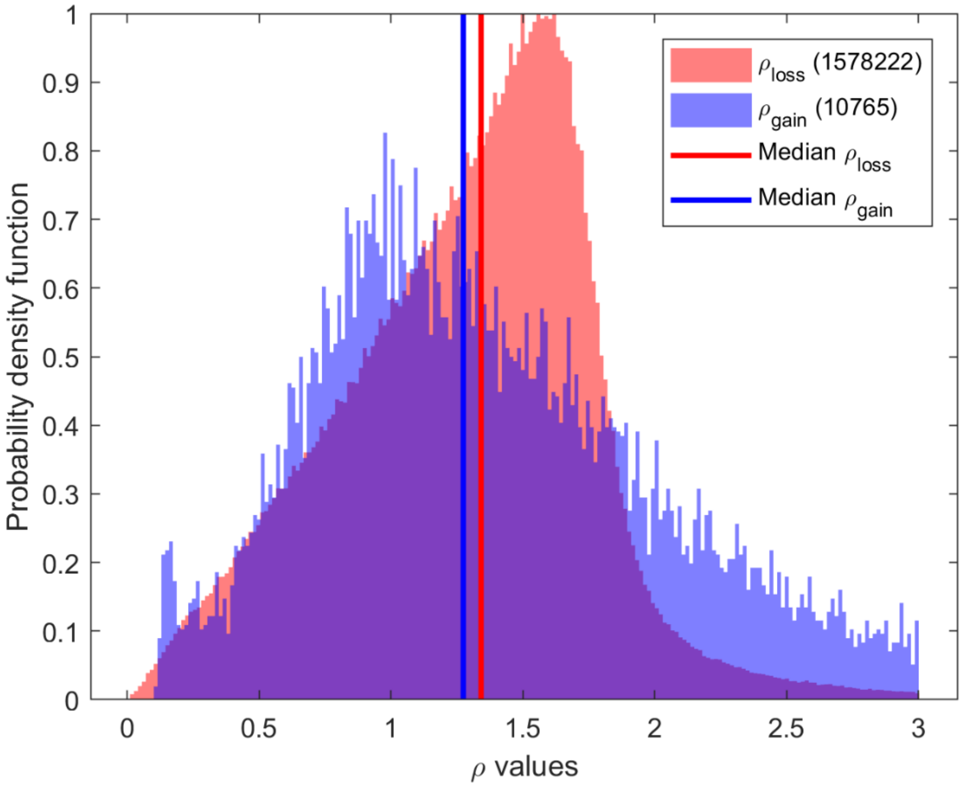

Figure 13. Density of biomass per percentage of tree cover. Probability density functions of density of biomass per percentage of tree cover lost (ρ_{loss}) and gained (ρ_{gain}) for Southern Finland. Number in brackets report the sample sizes.

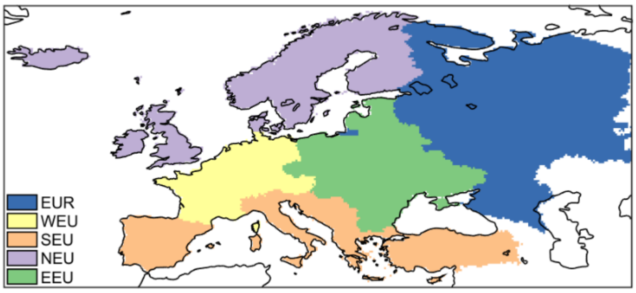

Figure 14. European regions. Grouping of countries in macro-areas shown in different colours. Eastern Europe (EEU) includes Belarus, Bulgaria, Czech Republic, Estonia, Hungary, Latvia, Lithuania, Poland, Republic of Moldova, Romania, Slovakia, and Ukraine. Northern Europe (NEU) includes Denmark, Iceland, Ireland, Finland, Norway, Sweden, and United Kingdom. Southern Europe (SEU) includes Albania, Bosnia and Herzegovina, Croatia, Cyprus, Greece, Italy, Kosovo, Malta, Montenegro, Portugal, Republic of Macedonia, Serbia, Slovenia, Spain, and Turkey. Western Europe (WEU) includes Austria, Belgium, France, Netherlands, Luxembourg, Germany, Liechtenstein, and Switzerland. European Russia (EUR) includes the Russian federation within the continental Europe.

References

1. Santoro, M. GlobBiomass - global datasets of forest biomass. (2018) doi:https://doi.org/10.1594/PANGAEA.894711.
2. Hansen, M. C. *et al.* High-Resolution Global Maps of 21st-Century Forest Cover Change. *Science* **342**, 850–853 (2013).
3. Simard, M., Pinto, N., Fisher, J. B. & Baccini, A. Mapping forest canopy height globally with spaceborne lidar. *J. Geophys. Res. Biogeosciences* **116**, (2011).
4. Poulter, B. *et al.* The global forest age dataset (GFADv1.0), link to NetCDF file. *NASA National Aeronautics and Space Administration* (2018) doi:https://doi.org/10.1594/PANGAEA.889943.
5. Glick, H. B. *et al.* Spatially-explicit models of global tree density. *Sci. Data* **3**, 160069 (2016).
6. Abatzoglou, J. T., Dobrowski, S. Z., Parks, S. A. & Hegewisch, K. C. TerraClimate, a high-resolution global dataset of monthly climate and climatic water balance from 1958–2015. *Sci. Data* **5**, 170191 (2018).
7. De Martonne, E. Une nouvelle fanction climatologique: l'indice d'aridité. *La Météorologie* **2**, 449–458 (1926).
8. Vicente-Serrano, S. M., Beguería, S. & López-Moreno, J. I. A Multiscalar Drought Index Sensitive to Global Warming: The Standardized Precipitation Evapotranspiration Index. *J. Clim.* **23**, 1696–1718 (2009).
9. Field, R. D. *et al.* Development of a Global Fire Weather Database. *Nat. Hazards Earth Syst. Sci.* **15**, 1407–1423 (2015).
10. Kanamitsu, M. *et al.* NCEP–DOE AMIP-II Reanalysis (R-2). *Bull. Am. Meteorol. Soc.* **83**, 1631–1644 (2002).
11. Pesaresi, M. *et al. Operating procedure for the production of the Global Human Settlement Layer from Landsat data of the epochs 1975, 1990, 2000, and 2014*. http://publications.jrc.ec.europa.eu/repository/handle/111111111/40182 (2016) doi:10.2788/253582 (online).
12. Kloster, S. *et al.* Fire dynamics during the 20th century simulated by the Community Land Model. *Biogeosciences* **7**, 1877–1902 (2010).
13. Pechony, O. & Shindell, D. T. Driving forces of global wildfires over the past millennium and the forthcoming century. *Proc. Natl. Acad. Sci.* **107**, 19167–19170 (2010).
14. Tuanmu, M.-N. & Jetz, W. A global, remote sensing-based characterization of terrestrial habitat heterogeneity for biodiversity and ecosystem modelling. *Glob. Ecol. Biogeogr.* **24**, 1329–1339 (2015).
