# Supplementary material for: Emergent vulnerability to climate-driven disturbances in European forests
Source: Nat Commun. 2021 Feb 23;12:1081. doi: 10.1038/s41467-021-21399-7 (PMC7902618; doi:10.1038/s41467-021-21399-7)
Supplement: Supplementary file 3 — Reporting Summary [file 41467_2021_21399_MOESM3_ESM.pdf]

nature research

Corresponding author(s): Giovanni Forzieri

Last updated by author(s): Jan 11, 2021

Reporting Summary

Nature Research wishes to improve the reproducibility of the work that we publish. This form provides structure for consistency and transparency in reporting. For further information on Nature Research policies, see our Editorial Policies and the Editorial Policy Checklist.

Statistics

For all statistical analyses, confirm that the following items are present in the figure legend, table legend, main text, or Methods section.

n/a	Confirmed	
☐	☒	The exact sample size (n) for each experimental group/condition, given as a discrete number and unit of measurement
☐	☒	A statement on whether measurements were taken from distinct samples or whether the same sample was measured repeatedly
☐	☒	The statistical test(s) used AND whether they are one- or two-sided<br>*Only common tests should be described solely by name; describe more complex techniques in the Methods section.*
☐	☒	A description of all covariates tested
☐	☒	A description of any assumptions or corrections, such as tests of normality and adjustment for multiple comparisons
☐	☒	A full description of the statistical parameters including central tendency (e.g. means) or other basic estimates (e.g. regression coefficient) AND variation (e.g. standard deviation) or associated estimates of uncertainty (e.g. confidence intervals)
☐	☒	For null hypothesis testing, the test statistic (e.g. F, t, r) with confidence intervals, effect sizes, degrees of freedom and P value noted<br>*Give P values as exact values whenever suitable.*
☒	☐	For Bayesian analysis, information on the choice of priors and Markov chain Monte Carlo settings
☒	☐	For hierarchical and complex designs, identification of the appropriate level for tests and full reporting of outcomes
☒	☐	Estimates of effect sizes (e.g. Cohen's d, Pearson's r), indicating how they were calculated

Our web collection on statistics for biologists contains articles on many of the points above.

Software and code

Policy information about availability of computer code

Data collection	R (version 4.0.1) and python (version 3.8.5) were used to collect the data.
Data analysis	MATLAB (version v2017b) was used to analyze the data and develop random forest models. Google Earth Engine was used to model biomass losses.

For manuscripts utilizing custom algorithms or software that are central to the research but not yet described in published literature, software must be made available to editors and reviewers. We strongly encourage code deposition in a community repository (e.g. GitHub). See the Nature Research guidelines for submitting code & software for further information.

Data

Policy information about availability of data

All manuscripts must include a data availability statement. This statement should provide the following information, where applicable:

- Accession codes, unique identifiers, or web links for publicly available datasets
- A list of figures that have associated raw data
- A description of any restrictions on data availability

The observation-driven datasets analyzed in this study are publicly available as referenced in the following lines:
Observed forest disturbances
Fires | European Forest Fire Information System https://effis.jrc.ec.europa.eu/
Windthrows | European Forest Windthrow dataset https://doi.org/10.6084/m9.figshare.9555008
Insect outbreaks | National Insect and Disease Survey http://foresthealth.fs.usda.gov

Forest features

April 2020

• Plant cover fractions https://www.esa-landcover-cci.org/
• Above ground biomass (biomass) https://globbiomass.org/products/global-mapping/
• Global Forest Change (GFC) maps https://earthenginepartners.appspot.com/science-2013-global-forest
• Tree height https://webmap.ornl.gov/wcsdown/dataset.jsp?ds_id=10023
• Tree age https://doi.pangaea.de/10.1594/PANGAEA.889943
• Leaf Area Index (LAI) https://doi.org/10.5067/MODIS/MCD15A3H.006
• NDVI values https://doi.org/10.5067/MODIS/MOD13Q1.006
• Tree density https://doi.org/10.6084/m9.figshare.3179986

Climate features
• Precipitation, average temperature and maximum temperature http://www.climatologylab.org/terraclimate.html
• Standardized precipitation evapotranspiration index https://www.ecmwf.int/en/forecasts/datasets/reanalysis-datasets/era5
• Fire Weather Index (FWI) https://data.giss.nasa.gov/impacts/gfwed/
• Wind speed (Wind speed) and cumulated snow (Snow) https://www.esrl.noaa.gov/psd/data/gridded/data.ncep.reanalysis2.html.

Landscape features
• Population density http://ghsl.jrc.ec.europa.eu/ghs_pop.php.
• Coefficient of spatial variation (CV), Evenness Index (Evenness) and Homogeneity Index (Homogeneity) http://www.earthenv.org/texture.
• Elevation and Slope https://www.usgs.gov/land-resources/eros/coastal-changes-and-impacts/gmted2010.

The generated vulnerability models are available at https://doi.org/10.6084/m9.figshare.13577960.
The custom MATLAB code written to analyze the data, develop the random forest models and generate figures is available at https://doi.org/10.6084/m9.figshare.13577960. Additional codes written in R/Python and Google Earth Engine used for data pre-processing are available on request from the corresponding author.

Field-specific reporting

Please select the one below that is the best fit for your research. If you are not sure, read the appropriate sections before making your selection.

☐ Life sciences ☐ Behavioural & social sciences ☒ Ecological, evolutionary & environmental sciences

For a reference copy of the document with all sections, see nature.com/documents/nr-reporting-summary-flat.pdf

Ecological, evolutionary & environmental sciences study design

All studies must disclose on these points even when the disclosure is negative.

Study description	We investigated the vulnerability of European forests to fires, windthrows and insect outbreaks over the period 1979-2018.The proposed methodology is based on a machine learning approach that is purely data-driven (Earth observations, climate drivers, database of forest disturbances) and therefore reproducible, applicable at large scales, and in line with the measurement/reporting/verification process of UNFCCC.
Research sample	In order to identify/calibrate/validate vulnerability models we used a large number of records of forest disturbances collected over the 2000-2017 period. Fires were retrieved from the European Forest Fire Information System (EFFIS, https://effis.jrc.ec.europa.eu/) and count 15,818 records. Windthrows were acquired from the European Forest Windthrow dataset (FORWIND, https://doi.org/10.6084/m9.figshare.9555008) with 89,743 records. Insect outbreaks were retrieved from the National Insect and Disease Survey (IDS, http://foresthealth.fs.usda.gov) database of the United States Department of Agriculture (USDA) which includes 50,777 records.
Sampling strategy	Potential effects of spatial dependence structure in the observational datasets were reduced by resampling target and predictor variables along the gradients of the three principal components (PC) derived from the initial set of predictors. To this aim, we used 20 bins of equal intervals for each PC dimension spanning the full range of values. The resampling procedure was stratified by splitting the records in training and testing sets. For each year between 2000 and 2017, we randomly extracted 60% of the records. The extracted subset was then binned in the PC space using the average as aggregation metric weighted by the areal extents of each disturbance record. The remaining 40% of records were similarly processed and used as independent validation set. The number of bins conditioned the size of the final samples used to calibrate and validate vulnerability models and it was chosen as a trade-off to reduce potential pseudo-replications and preserve the major sources of variability in the original records.
Data collection	A set of environmental variables of three major categories, including forest properties, climate and landscape features, were selected as potential predictors of forest vulnerability based on existing literature. These variables were retrieved from publicly available geo-spatial products, including satellite and reanalysis data (details in Methods and Supplementary Methods 1). Each variable was spatially averaged over the forest area of each disturbance record.
Timing and spatial scale	We explored the 1979-2018 period using monthly and annual temporal resolution of data, resampled to the common 0.25° spatial resolution for the Europe domain.
Data exclusions	In order to focus on effective damaging events in forest ecosystems, only records with relative biomass loss > 5% were selected. In the case of windthrows, we noted that maximum wind speeds retrieved from 0.5° spatial resolution of reanalysis data may largely underestimate effective maximum winds. This was particularly evident for tornado events, given their limited spatial extents compared to the grid cell, and the storm event Klaus that occurred in 2009 and for which we noticed an underestimation of the effective wind speed of the 78% (retrieved ~12 ms-1 instead of observed maximum wind speed of 55 ms-1). Therefore, such events were excluded from our analysis. The exclusion criteria were defined after preliminary data exploration.

Reproducibility	All computations are clearly described in the method section and can be reproduced. To ensure full reproducibility of our calculations, random forest generators (used in the random forest models) have been set using the same seed.
Randomization	For each disturbance type, we developed a random forest (RF) regression model to predict the observed relative biomass loss (response variable) based of pre-event environmental conditions (predictors). The use of machine learning in general and of RF in particular, being nonparametric and nonlinear data-driven methods, avoids making potentially strong assumptions about the functional form relating the key drivers and the response functions to natural disturbances. The RF implemented here uses 500 regression trees, whose depth and number of predictors to sample at each node were identified using Bayesian optimization.
Blinding	Our study does not deal with experiments that require blinding.

Did the study involve field work? ☐ Yes ☒ No

Reporting for specific materials, systems and methods

We require information from authors about some types of materials, experimental systems and methods used in many studies. Here, indicate whether each material, system or method listed is relevant to your study. If you are not sure if a list item applies to your research, read the appropriate section before selecting a response.

Materials & experimental systems

n/a	Involved in the study
☒	☐ Antibodies
☒	☐ Eukaryotic cell lines
☒	☐ Palaeontology and archaeology
☒	☐ Animals and other organisms
☒	☐ Human research participants
☒	☐ Clinical data
☒	☐ Dual use research of concern

Methods

n/a	Involved in the study
☒	☐ ChIP-seq
☒	☐ Flow cytometry
☒	☐ MRI-based neuroimaging
